# Atypical Hemolytic Uremic Syndrome-Associated FHR1 Isoform FHR1*B Enhances Complement Activation and Inflammation

**DOI:** 10.3389/fimmu.2022.755694

**Published:** 2022-01-21

**Authors:** Boyang Xu, Yuqi Kang, Yujing Du, Weiyi Guo, Li Zhu, Hong Zhang

**Affiliations:** ^1^ Renal Division, Department of Medicine, Peking University First Hospital, Peking University Institute of Nephrology, Key Laboratory of Renal Disease (Peking University), National Health Commission, Key Laboratory of Chronic Kidney Disease Prevention and Treatment, Ministry of Education, Beijing, China; ^2^ Department of Nuclear Medicine, Peking University First Hospital, Beijing, China

**Keywords:** complement, FHR1, complement activation, aHUS, inflammation

## Abstract

Atypical hemolytic uremic syndrome (aHUS) is a rare but severe type of thrombotic microangiopathy that is triggered by the abnormal activation of the alternative complement pathway. Previous studies have reported that three completely linked coding variants of *CFHR1* form two haplotypes, namely, CFHR1*A (c.469C, c.475C, c.523G) and CFHR1*B (c.469T, c.475G, c.523C). CFHR1*B is associated with susceptibility to aHUS. To explore the genetic mechanism by which *CFHR1* isoforms contribute to aHUS, we compared the structures of FHR1*A and FHR1*B by homology modeling and found differences in the angles between SCR3 and SCR4-SCR5, as FHR1*B had a larger angle than FHR1*A. Then, we expressed FHR1*A and FHR1*B recombinant proteins and compared their functions in complement system regulation and inflammation. We found that FHR1*B presented a significantly higher capacity for binding C3b and necrotic cells than FHR1*A. In a cofactor assay, the FHR-1*B showed stronger influence on FH mediated cofactor function than the FHR-1*A, resulted in fewer C3b cleavage products. In the C3 convertase assays, FHR1*B showed more powerful effect compared with FHR1*A regarding to de-regulate FH function of inhibition the assembling of C3bBb. Additionally, we also found that FHR1*B triggered monocytes to secrete higher levels of IL-1β and IL-6 than FHR1*A. In the present study, we showed that variants of *CFHR1* might differently affect complement activation and sterile inflammation. Our findings provide a possible mechanism underlying the predisposition to aHUS caused by *CFHR1* isoform CFHR1*B.

## Introduction

Atypical hemolytic uremic syndrome (aHUS) is a rare but severe type of thrombotic microangiopathy that mainly presents as a triad of microangiopathic hemolytic anemia (negative Coombs’ test results), thrombocytopenia, and acute renal failure (1); aHUS has a mortality rate of 25%, and half of the patients with aHUS progress to end-stage renal disease (2). In contrast to typical hemolytic uremic syndrome, which is caused by infection with Shiga toxin-producing *Escherichia coli (* 3), aHUS is mainly caused by abnormalities in the alternative complement pathway that trigger complement system overactivation and further cause endothelial cell damage and thrombosis (4, 5). Among all aHUS patients, approximately 60% have genetic abnormalities in complement pathway proteins (6, 7), and 20-30% of these genetic abnormalities are located in Complement factor H (FH) (2, 8).

FH is an important negative complement regulatory protein of the alternative pathway that can accelerate the decay of C3 convertase and C5 convertase and facilitate the cleavage of C3b by factor I (FI). In addition, humans have 5 genes (*CFHRs*) located downstream of the *CFH* gene that encode 5 complement FH-related proteins (FHR1, FHR2, FHR3, FHR4, and FHR5), which show high sequence similarity to FH (9, 10). Some complement FH-related proteins, including FHR1, FHR2 and FHR5, can compete with FH and dysregulate complement activation (11–14). Moreover, FHR1 was reported to bind to monomeric C-reactive protein and enhance complement activation (15). FHR-1 bound to late apoptotic and necrotic cells to enhance complement activation alone and in collaboration with monomeric CRP and pentraxin 3 (16). FHR1 was reported to modulate neutrophil functions independent of complement activation (17). More recently, Irmscher et al. reported the function of FHR1, namely, the induction of monocytic inflammation independent of complement (18, 19). Currently, the function of FHRs is still incompletely understood.

A variety of genetic abnormalities in *CFH* and *CFHRs*, including deletions, variants and hybrid genes (7), have been reported as predisposing factors of aHUS. Moreover, some common variants of *CFH* and *CFHRs* have been associated with susceptibility to aHUS. Recently, Abarrategui-Garrido et al. (20) reported three completely linked coding variants of *CFHR1* exon 4 (c.469 C>T, c.475 C>G, and c.523 G>C), which changed three amino acids in the SCR3 domain of FHR1 to form two FHR1 isotypes, namely, FHR1*B (p.157Tyr, p.159Val, p.175Gln; basic isoform) and FHR1*A (p.157His, p.159Leu, p.175Glu; acidic isoform). Furthermore, these authors also observed that CFHR1*B predisposed patients to susceptibility to aHUS (20). In addition, Anne Kopp, et al. found aHUS-associated FHR1*B variant showed reduced binding to PTX3 in comparison with FHR1*A (21).

Based on the genetic evidence about the role of *CFHR1* in aHUS, in this study, we investigated the structures and functions of the FHR1*A and FHR1*B proteins to explore the mechanism underlying the roles of these *CFHR1* isotypes in aHUS.

## Methods

### Homology Modeling of FHR1*A and FHR1*B

There was no complete crystal structure of the FHR1 protein in the protein data bank (PDB) database. We performed homology modeling of SCR3-5 of FHR1*A and FHR1*B by using the ModBase database (22) to predict the effects of coding variants of FHR1 on the structure of its encoded protein. The C-terminal region (SCR18-20) of FH (PDB accession code: 3SW0), which showed a high sequence identity with the C-terminal region of FHR1, was defined as the template structure, and the models of FHR1*A and FHR1*B were analyzed *via* PyMOL software version 1.7.0.0. The homology modeling results were further evaluated with the given quality criteria (23).

### Protein Expression and Purification of FHR1

The codon-optimized (*Homo sapiens*) DNA sequences for CFHR1*A and CFHR1*B (fused to a hexa-histidine tag at the C-terminus), which had been synthesized and ligated into the pTT5 vector, were customized and provided by Genescript. The expression vectors were then transiently transfected into 293F cells using polyethyleneimine according to the manufacturer’s instructions (PEI, Polysciences Inc.). Then, the 293F cells were cultured in serum-free HEK293 cell complete medium (Sino Biological Inc.) in an incubator with 5% CO_2_ at 37°C for four days. After centrifugation and filtering, the harvested cell supernatant was then applied to a Ni Sepharose column (GE Healthcare) to obtain the His-tagged FHR1 proteins. The purified FHR1*A and FHR1*B proteins were finally concentrated in PBS by using an Amicon Ultra Centrifugal Filter (Merck). After purification and concentration, FHR-1 proteins were stored in PBS in -80°C.

### Identification of the Expressed FHR1 Proteins

The recombinant FHR1*A and FHR1*B proteins were analyzed by Coomassie blue staining and Western blotting. Briefly, the FHR1 proteins were separated by SDS-polyacrylamide gel electrophoresis (SDS-PAGE), and then, the gels were stained with Coomassie blue staining solution. For Western blotting, after SDS-PAGE, the FHR1 proteins were transferred to polyvinylidene fluoride membranes (PVDF membranes, Millipore). After blocking, the membranes were immunoblotted with mouse anti-His tag (Origene) or mouse anti-human FHR1 antibody (R&D, cross-reacts with FH), followed by anti-mouse IgG-HRP antibodies (Santa Cruz). Finally, the membranes were developed with a chemiluminescent HRP substrate (Millipore) according to the manufacturer’s instructions.

### Binding of FHR1 to C3b

To assess the capacities of FHR1*A and FHR1*B to bind C3b, we conducted solid-phase C3b binding assays. Serial dilutions of the recombinant FHR1*A and FHR1*B proteins (2.5 μg/ml-0.078 μg/ml in PBS) were coated onto plates (Thermo Fisher Scientific) and incubated at 4°C for more than 16 hours. After washing with 0.1% PBST and blocking with 1%BSA/PBST at 37°C for 1 hour, C3b (2 μg/ml) was added and incubated at 37°C for 1 hour. Then, FHR1-bound C3b was detected using a C3c polyclonal antibody (Dako), followed by the addition of an anti-rabbit IgG-alkaline phosphatase antibody (Santa Cruz). In the reverse setting, 100nM C3b (Complement Tech) dissolved in TBS (140mM NaCl, 2mM CaCl_2_, 1mM MgCl_2_ and 10mM Tris, pH 7.4) were immobilized in the microtiter plates at 4°C overnight. After blocking with 3% milk in 0.02%TBST for 2 hours at 25°C, serially diluted FHR1-A and FHR1-B proteins (10μg/ml-1.25μg/ml) were added and incubated for 30min at 37°C. And mouse anti-human FHR1 antibody (R&D, cross-reacts with FH) was added as primary antibody, followed by adding AP-conjugated goat anti-mouse IgG antibody (Sigma-Aldrich). Finally, the plates were developed with alkaline phosphatase chromogenic substrate (Sigma-Aldrich), and the optical density was read at 405 nm.

### Binding of Surface-Bound C3b

Binding of FHR1 with surface-bound C3b was detected as previously reported (24). In brief, Saccharomyces cerevisiae strain (Mingzhoubio B47202) cells were resuspended in TBS and added in the microtiter plates 200μl per well for about 45min RT for deposition. After washing away the extra cells with 0.02%TBST and blocking with 3% milk in 0.02%TBST for 2 hours at 25°C, 100nM C3b or TBS were added and incubated for 1 hour at 37°C. And after incubation with serial dilution of FHR1*A and FHR1*B (5μg/ml-1.25μg/ml), mouse anti-human FHR1 antibody (R&D, cross-reacts with FH), and AP-conjugated goat anti-mouse IgG antibody (Sigma-Aldrich) were added for incubation in succession. In the last, alkaline phosphatase chromogenic substrate were added and the optical density was read at 405 nm.

### Binding of FHR1 to Necrotic Cells

The capacities of FHR1*A and FHR1*B to bind to necrotic cells were examined by flow cytometry using human umbilical vein endothelial cells (HUVECs) as previously reported (18, 25). HUVECs were purchased from ScienCell Corporation (ScienCell, Carlsbad, CA) and cultured according to the manufacturer’s protocols. Briefly, 10^6^ HUVECs/ml were incubated at 90°C for 10 min in 1%BSA/PBS and after blocking with 1%BSA/PBS, cells were then incubated with serial dilutions of the recombinant FHR1 proteins (FHR1*A or FHR1*B; 0.313 μg/ml-0.078 μg/ml in 1%BSA/PBS) at 37°C for 30 min. After washing with PBS, the HUVECs were stained with a mouse anti-human FHR1 antibody (JHD10, Hycult Biotech, cross-reacts with FHR2 and FHR5) or mouse IgG1 [CT6] isotype control (Abcam) followed by incubation with an Alexa Fluor^®^ 647-conjugated anti-mouse IgG antibody (Cell Signaling Technology) as the secondary antibody. The necrosis induction was measured by propidium iodide and Annexin V staining (BD556547). Double positive for both Annexin V and PI cells were determined as necrotic cells. Cell acquisition was performed by a FACScan flow cytometer (BD), 10,000 cells were measured for each sample and data were analyzed by FlowJo (v10).

### Competition ELISA Assay for FHR1 and FH for C3b

To compare the capacity of FHR1*A and FHR1*B to compete with FH to bind C3b, FH (9 μg/ml) was immobilized on the surface of a microtiter plate (Thermo Fisher Scientific) at 4°C overnight. After blocking with 1% BSA/PBST at 37°C for 1 hour, serial dilutions of the recombinant FHR1*A or FHR1*B proteins (2.5 μg/ml-0.039 μg/ml) as well as C3b (0.25 μg/ml) were added to the plates. After shaking for 30 sec, the plates were incubated at 37°C for 1 hour. After washing with 0.1%PBST, FH-bound C3b was detected with a C3c polyclonal antibody (Dako), followed by incubation with an alkaline phosphatase-conjugated anti-rabbit IgG antibody as the secondary antibody (Sigma-Aldrich). The plates were then developed with alkaline phosphatase chromogenic substrate (Sigma-Aldrich), and the optical density was read at 405 nm.

### Cofactor Assay

To investigate the effect of FHR1 on the cofactor activity of FH, the generation of C3b cleavage products by complement factor I (CFI) was assessed by SDS-PAGE and Western blotting. Two serially diluted isoforms of FHR1 (125 μg/ml-62.5 μg/ml), alone or in combination with FH (15.64 ng), were mixed with C3b (1.1 μg) and CFI (100 ng) and then incubated at 37°C for 30 min. After SDS-PAGE and Western blotting, the C3b degradation product (α’ 43 kDa) was detected with a C3c polyclonal antibody (Dako), followed by an HRP-conjugated anti-rabbit IgG antibody (Jackson ImmunoResearch Labs). The membranes were developed with a chemiluminescent HRP substrate (Millipore), and the signals of the C3b degradation product (α’ 43 kDa) were analyzed by ImageJ.

### Convertase Assay

To evaluate the potential effect of FHR1 on the FH-mediated regulation of the C3bBb, C3 convertase assay was performed as previously described (24). In brief, at first 5μg/ml C3b (Complement Tech) were immobilized in the microtiter plates. After blocking with 3% milk in 0.02%TBST for 2 hours at 25°C, 4μg/ml factor B (Millipore), 8μg/ml properdin (Millipore), 0.2μg/ml factor D (Millipore), 20μg/ml BSA (Sigma-Aldrich), altogether with 100nM factor H (Complement Tech) and serially diluted FHR1*A or FHR1*B (300nM-500nM) were added. After incubation, anti-factor B goat polyclonal antibody (Sigma-Aldrich) was added for incubation, followed by adding AP-conjugated rabbit anti-goat IgG antibody (Sigma-Aldrich). Finally, after incubated with alkaline phosphatase chromogenic substrate, the optical density was read at 405 nm.

### Stimulation of Inflammatory Cytokine Production of Monocytes by FHR-1

As previously reported (18), we evaluated the effect of FHR1 as a driver of monocytic inflammation. Briefly, peripheral blood mononuclear cells (PBMCs) were freshly isolated from the whole blood of three independent healthy donors by the density gradient centrifugation method using Ficoll Pague Plus (GE Healthcare Life Science). Then, the monocytes were indirectly magnetically labeled and isolated from the PBMCs using the Classic Monocyte Isolation Kit (Miltenyi Biotec). Then, 96-well microtiter plates (Thermo Fisher Scientific) were coated with recombinant-FHR1*A, FHR1*B, and BSA for 1 hour at 37°C. Then, 1x10^5^ monocytes were incubated in the plates with immobilized FHR1 in complete medium with 10% normal human serum (NHS) for 20 hours at 37°C in an incubator with 5% CO_2_. Finally, the levels of the inflammatory factors IL-1β and IL-6 in the supernatants after 20 hours of incubation were measured by enzyme-linked immunosorbent assay (ELISA) using IL-1β (R&D) and IL-6 (R&D) ELISA kits according to the manufacturers’ protocols.

### Statistical Analyses

Continuous variables with normal distribution are expressed as mean ± standard deviation and independent *t*-test or One way-ANOVA is used for comparison between groups. For non-normally distributed variables, the median and interquartile range (IQR) are used for description, while the Kruskal-Wallis test or Mann-Whitney U test is used for comparison. Statistics were performed using SPSS25.0 software and graphing were performed by GraphPad Prism 7.0 software. A two-tailed P value less than 0.05 was considered statistically significant.

## Results

### Modeling Evaluation and Analysis

**Figure 1:**
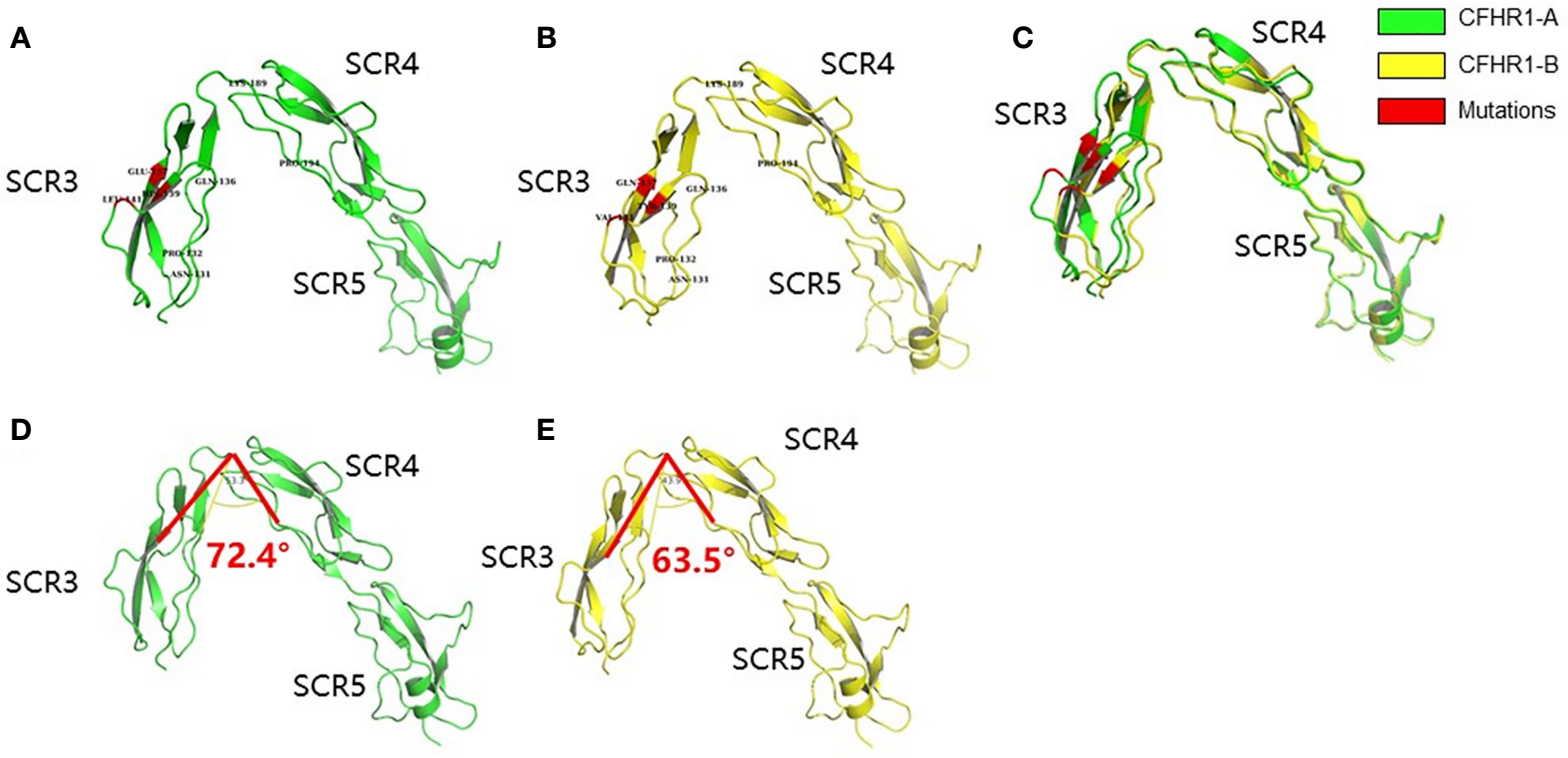
Schematic diagram of the homologous modeling structure of FHR1*A and FHR1*B. The SCR3-SCR5 of FHR1*A **(A)** and FHR1*B **(B)** are shown in green and yellow, respectively. The mutant amino acids are shown in red. The aligned view of SCR3-SCR5 of FHR1*A and FHR1*B showed some differences **(C)**. The structures of SCR4-SCR5 are identical between FHR1*A and FHR1*B, while SCR3 of FHR1*B is more prone to SCR4-5 than that of FHR1*A. The representative angle (∠p.139-p.189-p.194) of CFHR1*B **(E)** is smaller than that of CFHR1*A **(D)**, with a maximum angle difference of 8.9°.

The structural models of FHR1*A and FHR1*B were successfully established *via* Modbase, and we selected the most reliable models for further analysis according to the quality criteria provided by the database (**Figures 1A, B**). After aligning the models of FHR1*A and FHR1*B (**Figure 1C**), we discovered that the overall spatial structures were similar. Although the secondary structure of the β-fold in SCR3 was not altered by the amino acid changes at p.139 and p.157, the conformations of FHR1*A and FHR1*B were still slightly different due to the three different amino acids, and SCR3 of FHR1*B was more prone to SCR4-5 than that of FHR1*A. To better analyze the conformation, we set p.189Lys as the vertex and p.189Lys-p.194Pro as the edge to measure the angles formed by different amino acids in SCR3 (**Table 1**). The angles in FHR1*B are generally smaller than those in FHR1*A, ranging from 6.3° to 8.9° (**Figures 1D, E**).

**Table 1:** The included angles of SCR3 to SCR4-5 in FHR1*A and FHR1*B.

Amino acids	FHR1*A	FHR1*B	Differences
p.139His (FHR1*A)/Tyr (FHR1*B)	72.4	63.5	8.9
p.141Leu (FHR1*A)/Val (FHR1*B)	80.6	73.3	7.3
p.157Glu (FHR1*A)/Gln (FHR1*B)	81.7	75.4	6.3

p.189Lys was set as the vertex and p.189Lys- p.194Pro was set as the edge to measure the angles formed by different amino acids in SCR3. The differences in the included angles in FHR1*A and FHR1*B range from 6.3° to 8.9°.

### Western Blotting Analysis of the Recombinant FHR1 Proteins

We expressed recombinant FHR1*A and FHR1*B proteins with His-tag in eukaryotic expression system and purified by Ni Sepharose affinity chromatography. On SDS-PAGE gels, the recombinant FHR1*A and FHR1*B proteins showed two clear bands at approximately 40 kDa, which were detected with the His-tag antibody and FHR1 monoclonal antibody; these results were consistent with two previously reported glycosylated forms of FHR1 (42 kDa and 37 kDa, **Supplemental Figure 1**) (26). Thereafter, the recombinant FHR1*A and FHR1*B proteins were used in the following experiments.

### FHR1*B Exhibited Increased Binding to C3b Compared With FHR1*A

**Figure 2:**
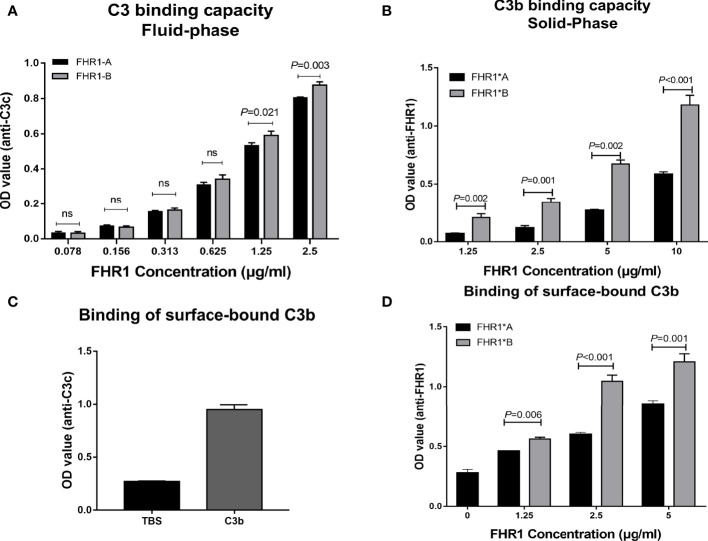
Interaction of the two FHR1 isoforms with C3b. Equal concentrations of two immobilized isoforms of FHR1 (0.078 μg/ml-2.5 μg/ml) bound C3b (2 μg/ml) in a dose-dependent manner. The binding-C3b was finally detected. FHR1*B showed a higher capacity to bind C3b at a concentration of 1.25 μg/ml **(A)**. In a reverse setting, equal concentrations of two isoforms of FHR1 (1.25 μg/ml-10 μg/ml) bound immobilized C3b (100nM) in a dose-dependent manner. The bound FHR1 was detected at last. FHR1*B also showed higher binding capacity to C3b **(B)**. Moreover, the 100nM C3b were added onto the microplates, overspread with the *Saccharomyces cerevisiae*, and equal concentrations of two isoforms of FHR1 (1.25 μg/ml-5 μg/ml) was added. The surface-bound C3b was detected by anti-C3c antibody **(C)**, and the binding of FHR1 to surface-bound C3b was detected with anti-FHR1 antibody **(D)**. The representative figures from three independent experiments are shown. The results are representative of three independent experiments. The data are presented as the means ± SDs. The symbol ns indicates nonsignificance.

Since the C-terminus of FHR1 showed 98% sequence identity with that of FH and the C-terminus of FH contained a C3b binding site, we explored the capacity of the two isoforms of FHR1 to bind to C3b. In the binding assay, both isoforms, namely, FHR1*A and FHR1*B, bound C3b in a dose-dependent manner. Moreover, at the same concentrations (either 1.25 μg/ml or 2.5 μg/ml), FHR1*B exhibited significantly higher C3b binding capacity than FHR1*A (FHR1*B vs. FHR1*A: at 1.25 μg/ml concentration, *P*=0.021; at 2.5 μg/ml concentration, *P*=0.003, **Figure 2A**). In the reverse setting, dose-dependent binding of the FHR-1 isoforms to immobilized C3b was measured, and FHR1*B also showed higher capacity of binding to C3b than FHR1*A (FHR1*B vs. FHR1*A: at 1.25μg/ml concentration, *P*=0.002; at 2.5μg/ml concentration, *P*=0.001; at 5μg/ml concentration, *P*=0.002; at 10μg/ml concentration, *P*<0.001, **Figure 2B**).

### FHR1*B Exhibited Increased Binding to Surface-Bound C3b Compared With FHR1*A

In surface-bound C3b binding assay, in which C3b was bound to Saccharomyces cerevisiae strain, we found that the FHR1*B isotype also has a stronger binding ability than FHR1*A to surface-bound C3b. (FHR1*B vs. FHR1*A: at 1.25μg/ml concentration, *P*=0.006; at 2.5μg/ml concentration, *P*<0.001; at 5μg/ml concentration, *P*=0.001, **Figures 2C, D**).

### FHR1*B Exhibited Increased Binding to Necrotic HUVECs Compared With FHR1*A

**Figure 3:**
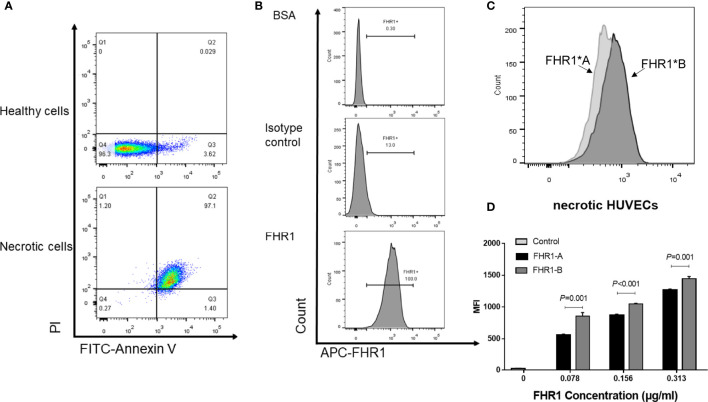
Binding of the FHR1 isoforms to necrotic cells. Necrosis cells was evaluated by PI and Annexin V staining. Double positive for both Annexin V and PI cells were considered as necrotic cells **(A)**. The representative figures for blank control, isotype control and sample incubated with FHR1 protein were showed **(B)**. FHR1 proteins (0.078 μg/ml-0.313 μg/ml) bound to necrotic HUVECs in a dose-dependent manner. The representative figures for samples incubated with both FHR1*A and FHR1*B protein were showed **(C)**. In addition, compared to FHR1*A, FHR1*B presented significantly increased capacity to bind to necrotic HUVECs at concentrations between 0.078 μg/ml and 0.313 μg/ml **(D)**. The results are representative of three independent experiments. The data are presented as the means ± SDs. MFI, Mean fluorescence intensity.

FHR1 was previously reported to bind to necrotic cells (13); therefore, we compared the capacity of the FHR1 isoforms to bind to necrotic HUVECs. We observed that both FHR1 isoforms bound to necrotic HUVECs (**Figure 3**) in a dose-dependent manner. In addition, at the same concentrations (0.313 μg/ml-0.078 μg/ml), FHR1*B showed significantly increased capacity to bind to necrotic HUVECs compared with FHR1*A (FHR1*B vs. FHR1*A: at 0.313 μg/ml concentration, *P*=0.001; at 0.156 μg/ml concentration, *P*<0.001; at 0.078 μg/ml concentration, *P*=0.001).

### FHR1*B Exhibited Increased Competition With FH for C3b Compared With FHR1*A

**Figure 4:**
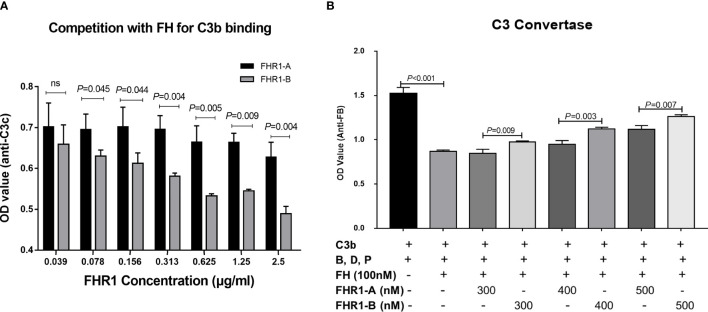
Competition assays of FHR1 with FH to C3b and C3b convertase assays. FHR1*A and FHR1*B compete with FH for binding C3b in a dose-dependent manner, and FHR1*B binds more C3b than FHR1*A at concentrations of 0.078 μg/ml-2.5 μg/ml **(A)**. FH inhibited the C3bBb assembling, while both isoforms of FHR1 competed with FH and reverse the inhibition to some extent. Moreover, FHR1*B showed a more powerful deregulation effect on FH mediated regulation of the solid-phase C3 convertase **(B)**. The results are representative of three independent experiments. The data are presented as the means ± SDs.

Since FHR1 was found to function as a competitive antagonist of FH to modulate activation of the complement cascade (27), we next explored whether FHR1*A and FHR1*B showed different levels of competition with FH. First, we evaluated the competition of FHR1*A and FHR1*B with FH for binding C3b. We found that FHR1*A and FHR1*B competed with FH for binding C3b in a dose-dependent manner, which was similar to previously reported observations of FHR1 (27). Moreover, within the concentration range of 0.078 μg/ml to 2.5 μg/ml, FHR1*B exhibited stronger competition with FH for binding C3b than FHR1*A, as FHR1*B decreased the binding by 10.49% (0.078 μg/ml FHR-1) and 30.52% (2.5 μg/ml FHR-1), while FHR1*A decreased the binding by only 1.11% and 10.73% (**Figure 4A**).

**Figure 5:**
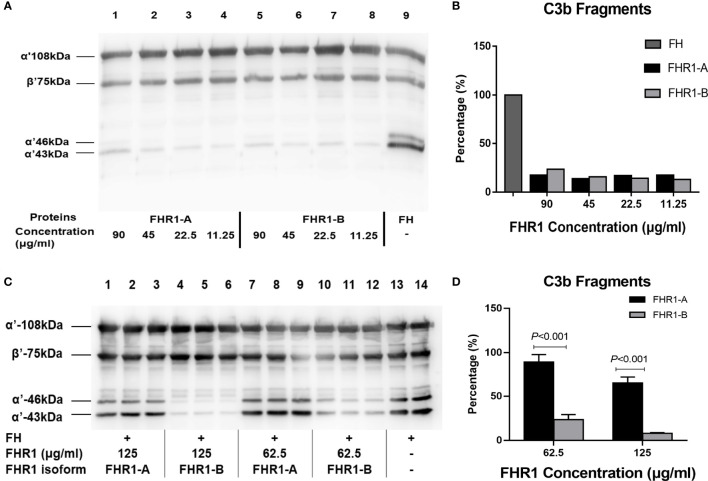
Effect of the FHR1 isoforms on the cofactor activity of FH. Compared to FH (positive control, lane 9), both FHR1*A (lanes 1-3) and FHR1*B (lanes 4-6) at concentrations of 90-11.25 ug/ml showed no or little FI cofactor activity **(A)**. When incubated with C3b, FI and FH together, the FHR1 groups (lanes 1-3, 4-6, 7-9, 10-12 were technical replicates, respectively; **(C)** generated fewer C3b cleavage products than the group without FHR1 (no-FHR1 control, lanes 13-14, technical replicates; **(C)**. Furthermore, the FHR1*B groups generated more C3b cleavage products than the FHR1*A groups at the same concentrations **(C, D)**. The data are presented as the means ± SDs.

Next, we performed a cofactor assay to assess the deregulation of FHR1 by FH in terms of its CFI cofactor activity. As previously reported, unlike FH, FHR1 showed no intrinsic cofactor activity for CFI (**Figures 5A, B**). Here, we observed that FHR1 competed with FH in a dose-dependent manner, which led to a reduction in the CFI cofactor activity of FH. At the same concentrations (either 125 μg/ml or 62.5 μg/ml), FHR1*B showed a higher capacity for de-regulating FH-mediated CFI cofactor activity than FHR1*A (FHR1*B vs. FHR1*A: at 62.5 μg/ml concentration, *P*<0.001; at 125 μg/ml concentration, *P*<0.001; **Figures 5C, D**).

### FHR1*B Exhibited Higher Deregulation Effect on FH Mediated Regulation of the Solid-Phase C3 Convertase

Given the negative regulation of FH on C3 convertase formation into consideration, we investigated the potential different effect of FHR1*B and FHR1*A on FH mediated regulation of the solid-phase C3 convertase. We found that FH inhibited the C3bBb assembling, while both isoforms of FHR1 competed with FH and reversed the inhibition. Moreover, FHR1*B showed more powerful deregulation effect on FH than FHR1*A, regarding the FH mediated regulation of the solid-phase C3 convertase. (FH vs. TBS, *P*<0.001. FHR1*B vs. FHR1*A: at 300nM concentration, *P*=0.009; at 400nM concentration, *P*=0.005; at 500nM concentration, *P*=0.007, **Figure 4B**).

### FHR1*B Induced Elevated Secretion of Higher IL-1β and IL-6 Than FHR1*A

**Figure 6:**
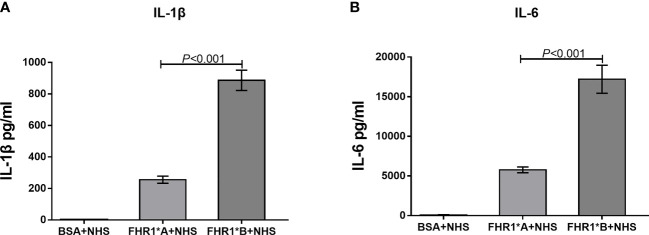
FHR1 induces monocytes to secrete inflammatory cytokines. Compared to BSA, both FHR1*A and FHR1*B significantly increased IL-1β **(A)** and IL-6 **(B)** secretion by monocytes. In addition, FHR1*B induced the secretion of higher levels of IL-1β **(A)** and IL-6 **(B)** by monocytes than FHR1*A. The data are presented as the means ± SDs. The results are representative of three independent experiments. The data are presented as the means ± SDs.

Recently, FHR1 was reported to play a proinflammatory role. We therefore compared the effects of FHR1*A and FHR1*B on monocytes. We found that monocytes incubated with FHR1*B secreted higher levels of IL-1β and IL-6 than monocytes incubated with FHR1*A. (FHR1*B vs. FHR1*A: IL-1β, *P*<0.001; IL-6, *P*<0.001; **Figure 6**).

## Discussion

aHUS is a rare variant of thrombotic microangiopathy that is triggered by abnormal alternative complement pathway activation, which leads to complement deposition on endothelial cells and causes endothelial cell swelling and detachment (28). In the present study, we showed that the *CFHR1* isoform CFHR1*B, which increases susceptibility to aHUS, has higher capacity for binding to C3b and could induce more inflammation than CFHR1*A, which suggested the involvement of *CFHR1* in aHUS.

*CFHR1* is a member of the *CFH* gene family, and its encoded product FHR1 is composed of 5 SCR domains. Three C-terminal SCRs (SCR3-5) of FHR1 show high sequence similarity with SCR18-20 of FH. The crystal structure of FH SCR18-20 was determined to be a ‘‘J’’-shape, in which SCR18 folds back toward SCR19. This structural characteristic of FH SCR18-20 suggested the presence of conformational mobility between SCR18 and SCR19 and little flexibility between SCR19 and SCR20 (29). FHR1*A and FHR1*B have three different amino acids in the SCR3 regions. To determine whether these three coding variants affected the protein structure of FHR1, we compared FHR1*A and FHR1*B by homology modeling and determine the differences in the angles between SCR3 and SCR4-SCR5. Therefore, we hypothesized that these angle differences might affect the protein flexibility of FHR1 and indirectly affect the function of FHR1 SCR4-SCR5.

Although only a few studies have focused on FHR1 SCR4-SCR5, its homologous motif in FH, SCR19-SCR20, has been widely investigated. Increasing evidence shows that FH SCR19 and SCR20 contain binding sites for C3b, glycosaminoglycans, and endothelial cells (30–32). Moreover, the majority of aHUS-associated FH mutations cluster within SCR19-SCR20 (7), supporting its interaction with endothelial cells. Similarly, FHR1 also has the ability to bind C3b and the surface of necrotic HUVECs (13, 18). To verify our hypothesis that the changes in the FHR1 structure induced by coding variants might indirectly influence the function of FHR1 SCR4-SCR5, we compared the capacities of FHR1*A and FHR1*B to bind C3b (including surface-bound C3b) as well as to necrotic HUVECs. Our results indicated that FHR1*B showed significantly higher capacity to bind to not only C3b (including surface-bound C3b) but also necrotic HUVECs. Our findings indicated that the coding variants of SCR3 may affect the function of FHR1, such as its binding C3b and necrotic cells.

In addition, FHR1 was reported to function as a competitive antagonist of FH (27). FH is a vital regulatory protein of complement in plasma that inhibits complement activation in two ways: interfering with the assembly and facilitating the decay of C3/C5 convertases by binding to C3b and acting as a cofactor to facilitate the cleavage of C3b by CFI (33). Therefore, we next evaluated the function of FHR1*A and FHR1*B in the de-regulation of FH. We found that both FHR1*A and FHR1*B could compete with FH to influence C3b binding, decrease the CFI-mediated cleavage and inactivation of C3b, and regulate C3 convertase formation in a dose-dependent manner, and these results are consistent with the FH competitor function of FHR1 (12, 13, 27). Compared to FHR1*A, FHR1*B presented an increased capacity to compete with FH, as shown by the generation of fewer FI-mediated cleavage products of C3b and more formation of C3 convertase, which would enhance complement activation. Our results indicated a stronger effect of FHR1*B than FHR1*A in the deregulation of FH.

Moreover, Irmscher et al. recently reported that FHR1 bound to necrotic-type cells and activated monocytic inflammasomes, and proved the FHR1 induced inflammation was independent of complement (15). In the present study, we also observed that FHR1*B has more powerful effects on triggering inflammation *via* monocytes, indicating that FHR1*B may cause damage in patients with aHUS through enhancement of both complement activation and inflammation.

However, our study had some limitations. Firstly, FHR-1 can form homodimers and heterodimers in circulation. However, lack of complete crystal structure of the FHR1 protein hindered us to further investigate the structural difference of FHR1*A and FHR1*B on dimers. Secondly, we didn’t evaluate the differences regarding complement activation and inflammation in aHUS patients with homozygous FHR1*A and FHR1*B in the present study. Future studies focused on genotype-phenotype correlation in large aHUS cohort will be needed. Thirdly, in the cofactor assay, the concentration of FHR1 to show effective competition for FH regarding the cofactor function is higher than physiologic concentration in circulation. Although local concentration of FHR3 and FHR4 in AMD patients were reported as much higher than in circulation (34), we are still lack of similar information about local FHR1 concentration.

In summary, our study showed that coding variants of *CFHR1* (c. C469T, c. C475G, c. G523C) might change the protein structure of FHR1, thereby influencing the FH de-regulation and proinflammatory functions of FHR1 and therefore influencing complement activation and inflammation. Our findings provide a possible genetic mechanism underlying the predisposition to aHUS caused by the *CFHR1* isoform CFHR1*B.

## Data Availability Statement

The raw data supporting the conclusions of this article will be made available by the authors, without undue reservation.

## Ethics Statement

The studies involving human participants were reviewed and approved by the ethics committee of Peking University First Hospital. The patients/participants provided their written informed consent to participate in this study.

## Author Contributions

LZ and HZ conceived and designed the study. BX, YK, and WG carried out the experiments and analyzed the data. BX, YK, and LZ wrote the manuscript. All authors have read and approved the manuscript.

## Funding

This project was supported by grants from the National Science Foundation of China (81922013, 81970598, 82070733), National Science Foundation of Beijing (7192209, 7202206), National Key Research and Development Program of China (2020YFC2005003), Beijing Science and Technology Plan Project of China (D181100000118003, Z161100000516005) and Chinese Academy of Medical Sciences Research Unit-Peking University (No. 2019RU023).

## Conflict of Interest

The authors declare that the research was conducted in the absence of any commercial or financial relationships that could be construed as a potential conflict of interest.

## Publisher’s Note

All claims expressed in this article are solely those of the authors and do not necessarily represent those of their affiliated organizations, or those of the publisher, the editors and the reviewers. Any product that may be evaluated in this article, or claim that may be made by its manufacturer, is not guaranteed or endorsed by the publisher.
