# Supplementary material for: Atypical Hemolytic Uremic Syndrome-Associated FHR1 Isoform FHR1*B Enhances Complement Activation and Inflammation
Source: Front Immunol. 2022 Jan 21;13:755694. doi: 10.3389/fimmu.2022.755694 (PMC8814109; doi:10.3389/fimmu.2022.755694)

Supplemental Figure Legends

Supplemental Figure 1. Verification of recombinant FHR1*A and FHR1*B proteins. Recombinant proteins were analyzed by Western blot using two different antibodies, anti-His tag (A) or anti-FHR1 (B), and by Coomassie blue staining (C) to recognize recombinant FHR1 proteins, respectively. Two equal, clear bands near 40kDa were found, representing two glycosylation forms of FHR1 (42kDa and 37kDa).

Supplemental Figure 1

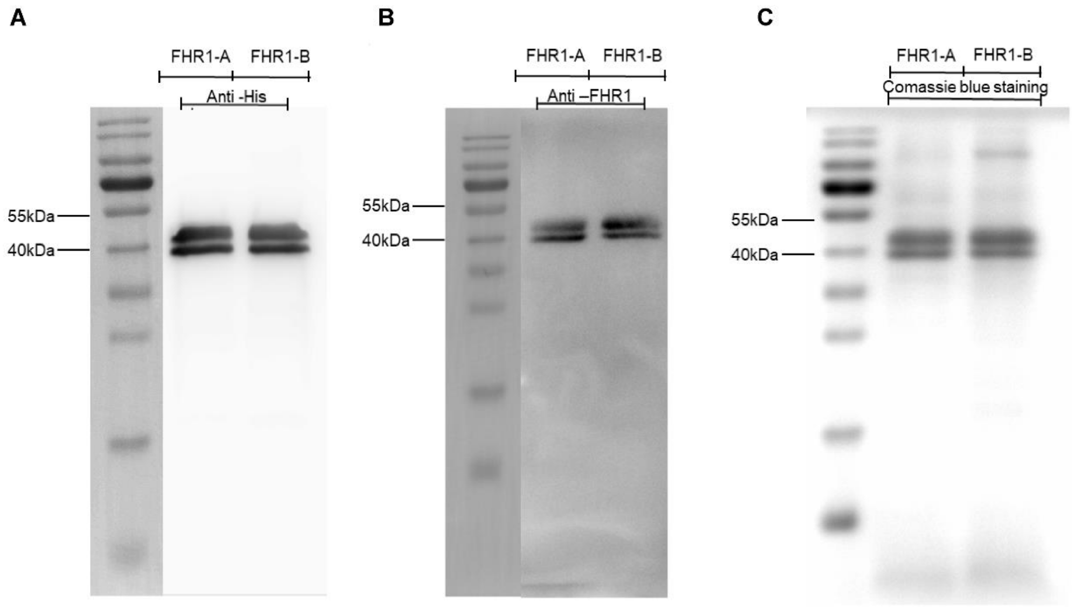

Supplement: Supplementary file 1 [file Image_1.pdf]
